# CIRCUS: a package for Circos display of structural genome variations from paired-end and mate-pair sequencing data

**DOI:** 10.1186/1471-2105-15-198

**Published:** 2014-06-18

**Authors:** Delphine Naquin, Yves d’Aubenton-Carafa, Claude Thermes, Maud Silvain

**Affiliations:** 1 Plateforme Intégrée IMAGIF – CNRS, Avenue de la Terrasse, Gif sur Yvette 91198, France; 2 Centre de Génétique Moléculaire - CNRS, Avenue de la Terrasse, Gif sur Yvette 91198, France

**Keywords:** Circos, Genomic structural variants, Genomic data visualization

## Abstract

**Background:**

Detection of large genomic rearrangements, such as large indels, duplications or translocations is now commonly achieved by next generation sequencing (NGS) approaches. Recently, several tools have been developed to analyze NGS data but the resulting files are difficult to interpret without an additional visualization step. Circos (Genome Res, 19:1639–1645, 2009), a Perl script, is a powerful visualization software that requires setting up numerous configuration files with a large number of parameters to handle. R packages like RCircos (BMC Bioinformatics, 14:244, 2013) or ggbio (Genome Biol, 13:R77, 2012) provide functions to display genomic data as circular Circos-like plots. However, these tools are very general and lack the functions needed to filter, format and adjust specific input genomic data.

**Results:**

We implemented an R package called CIRCUS to analyze genomic structural variations. It generates both data and configuration files necessary for Circos, to produce graphs. Only few R pre-requisites are necessary. Options are available to deal with heterogeneous data, various chromosome numbers and multi-scale analysis.

**Conclusion:**

CIRCUS allows fast and versatile analysis of genomic structural variants with Circos plots for users with limited coding skills.

## Background

NGS has become a widely used tool for detecting large-scale genome variations. When genomic DNA is to be sequenced, DNA is first fragmented. Genomic libraries can then be produced and sequenced from one end or both ends of the fragments, commonly referred to as single end or paired-end sequencing, respectively. Paired-end or mate-pair sequencing strongly facilitates the detection of genomic rearrangements and is therefore the preferred method for this type of analysis. Two reads of a fragment that align to abnormal positions on a chromosome, or to two different chromosomes, may indicate a structural variation. A list of these variations is difficult to analyze since one variation often joins two positions that were originally remote. There is no genome browser that permits visualization of these distant genomic events. Visualization tools [1-3] have been developed, displaying each variation as a link between positions on a circular ideogram. The most commonly used one, Circos, is very flexible but requires installation of Perl modules, a familiarity with the operating system to run effectively and with the parameters that are used in its configuration files. To circumvent this last difficulty, the variant detection program SVDetect [4] provides a script that converts its output into a format readable by Circos together with a limited tutorial set of configuration files. Recently, the RCircos package [5] was proposed to obtain Circos-like plots in an R environment [6]. However, the user cannot zoom into a chromosome and has to program the functions needed to generate input data. Circos, ggbio and RCircos are very powerful tools; however they were designed to manage a wide variety of analyses, and this flexibility leads to rather complex handling.

In order to provide fast visual analyses of structural genome variations, we have developed a wrapper of Circos for the R langage which supports a subset of Circos functionnalities and shelters the user from managing the large number of parameters in Circos configuration files. This software, CIRCUS, can parse output files from several variant structure detection tools to write all necessary files for Circos execution, customizable with options for a quick and flexible image production.

## Implementation

**Figure 1:**
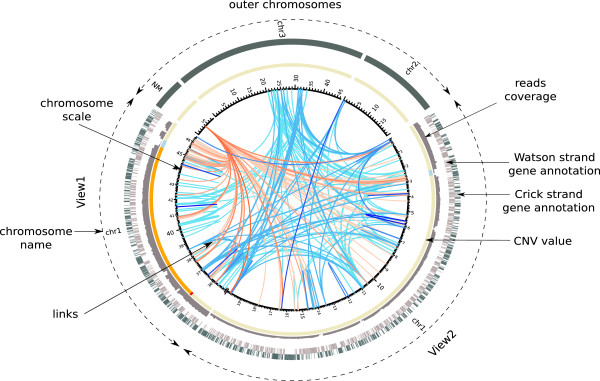
**CIRCUS image architecture.** NM links are colored in a red gradient, links resulting from SVDetect are colored in a blue gradient. Darker colors correspond to more significant links, supported by a high number of paired-end or mate-pair reads. For CNV track (resulting from Control-FREEC), background is in light yellow, regions of CNV value 0 are in ligtht blue, regions of value 2 are in orange and regions of value 3 are in red (results from Control-FREEC).

Besides the positions and the significance of the structural rearrangements, additional data can be informative on the final image display: local coverage in reads, Copy Number Variation (CNV) inference or/and gene annotations. CIRCUS can display all these features on a fixed framework that consists of 3 optional concentric rings; the most central ring represents the CNV inference in colored segments, the middle ring represents the read coverage in histogram style while the outer ring displays the genomic annotations in colored boxes (see Figure 1).

The concentric rings can be divided into two main parts. The first one contains one or two regions called “view(s)” within a chromosome of interest. The second was designed to display a set of entire chromosomes as well as an optional pseudo-chromosome (referred to as NM for “No Match” chromosome). It can be used to display the links for which one of the two reads does not map on the reference genome, what can indicate an integration site of a foreign DNA fragment. The relative size of these two parts can be adjusted. Inside the inner ring of the image, links are painted with color gradients according to the user defined values. Only links with at least one foot in the view(s) will be displayed.

**Figure 2:**
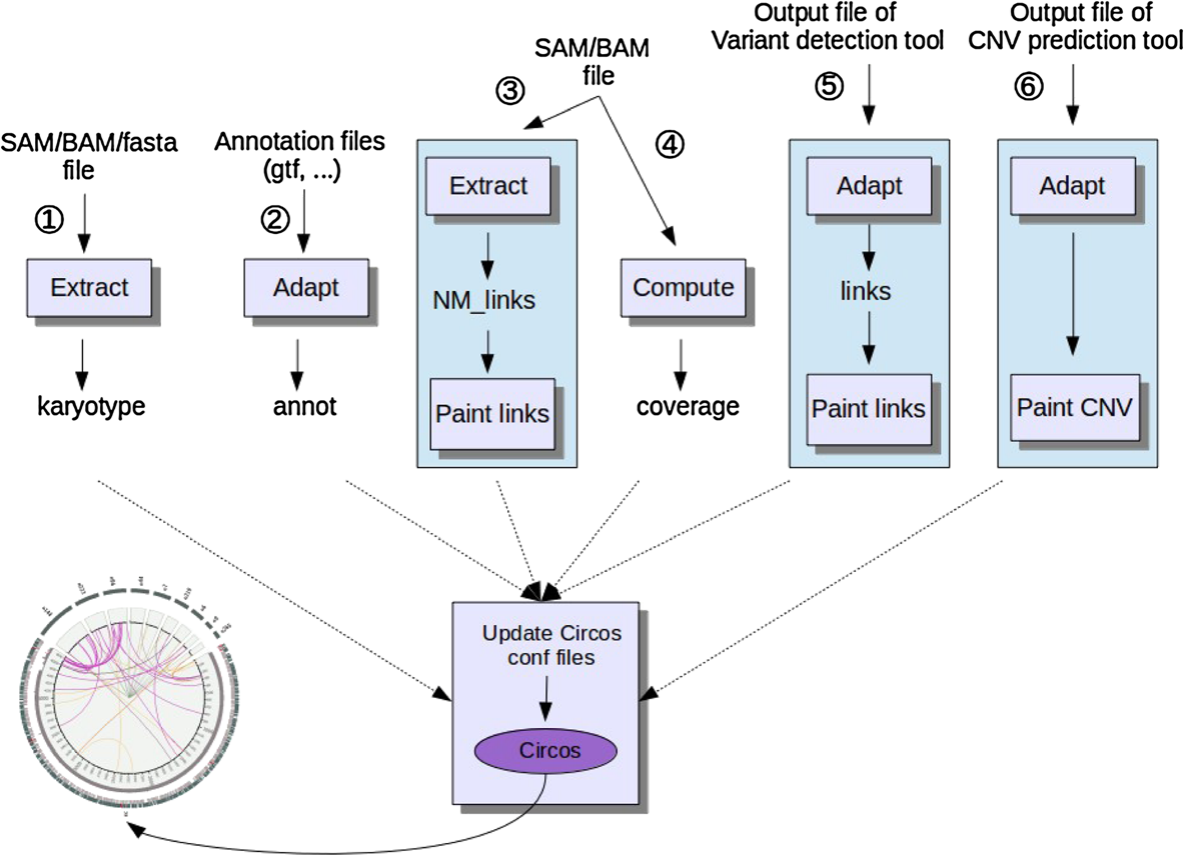
CIRCUS workflow.

The core of CIRCUS is an R function that allows the user to specify the dimensions of the components and the format of the picture (PNG or SVG), the type of features to be displayed, the chromosome coordinates of the region(s) to be analyzed and the chromosome orientation. According to the input parameters, this function filters the links and creates the configuration files required by Circos. To feed the core, as illustrated by Figure 2, data from gene annotation (pathway 2), NM links (pathway 3), reads coverage (pathway 4), intra-genomic links (pathway 5) and CNV status (pathway 6) have to be formatted, filtered, scaled and colored according to the user’s preferences. First, the karyotype function (pathway 1) can extract data from an input file to build a karyotype file. Then, five specific functions are provided to adapt the data format issued from different prediction tools to a CIRCUS format. Currently, genomic annotations from tabular format and intra-genomic links issued from SVDetect or Pindel [7] can be parsed. The writing of new converters from other software results is easy for programmers. In order to reduce computing time, reads coverage and NM links are computed from SAM files with internal calls to SAMtools/BEDtools packages and to a Python script. The third step, consisting in scaling and coloring the different features, is performed by four other functions which allow to display status, density, significance or other user-defined values. All these functions are linked in the data flow diagram depicted in Figure 2.

The core and peripheral functions have many input arguments to allow flexibility; to simplify their use, almost all have default values. At the end of the process, a log file is created showing all arguments used in function calls and the primary data used for the image display.

The aims of CIRCUS are to focus on structural genome variations, and to allow non-bioinformaticians to visualize their data in a straightforward way. Therefore, CIRCUS is an R wrapper that uses only a part of Circos functionalities. Ideogram skeleton components (thickness, ticks) are fixed, as well as most of the tracks graphic parameters, depending on the kind of data displayed: coverage is drawn in histogram style, CNVs in heatmap style, annotations in highlights style and links are simple lines with a fixed thickness. In this fixed framework, the user can decide which tracks to display and can set up zoom criteria. A typical analysis may include an iterative view of the links from each chromosome against all others, followed by zooms on regions of interest. The borders of each event can thus be precisely delineated, whatever the size of the corresponding DNA fragment. Localization of foreign sequence insertions such as mobile elements can be detected by links to the NM pseudo-chromosome.

## Result and discussion

**Figure 3:**
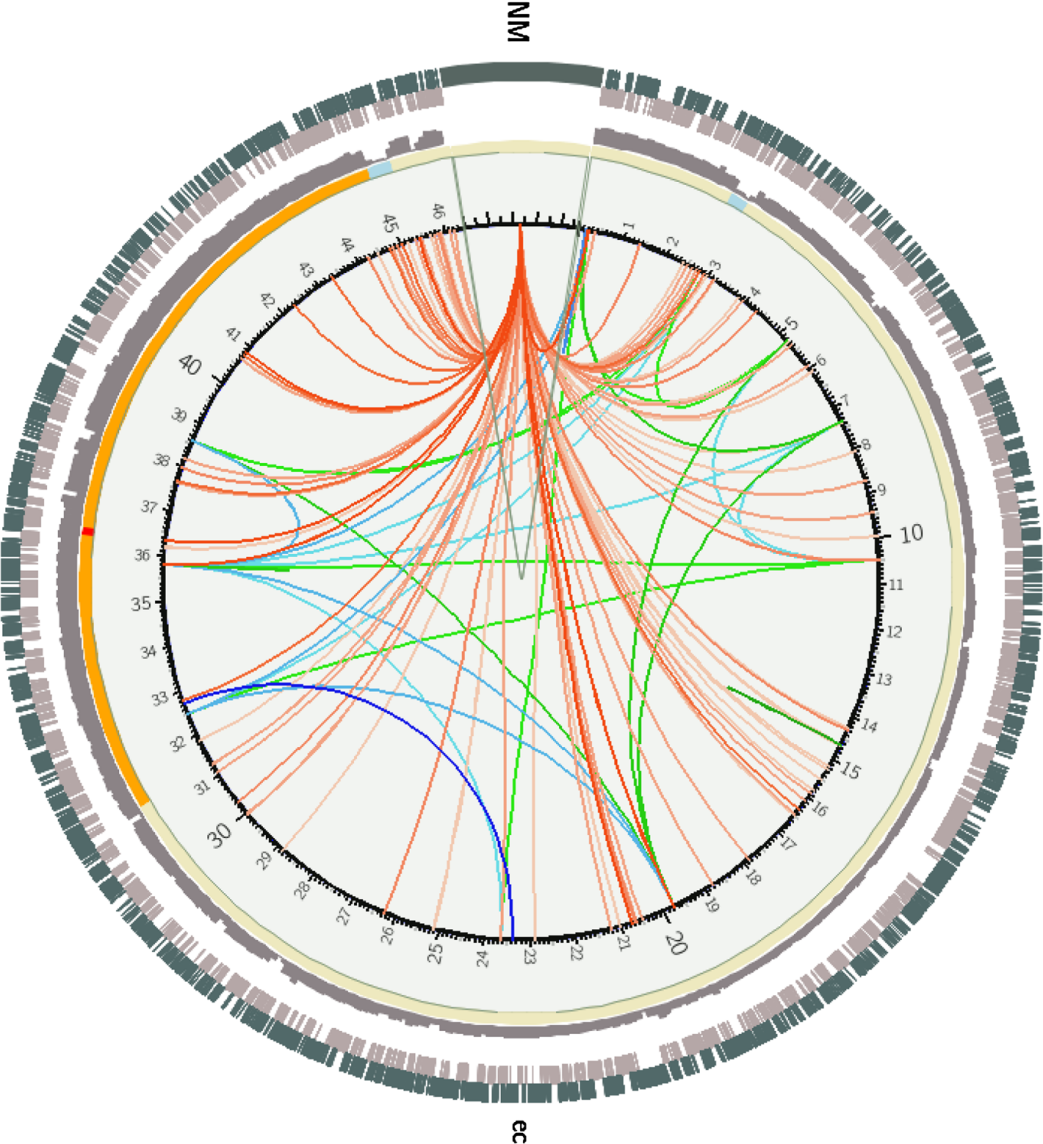
**Display of both INS_FRAGMT and INV_INS_FRAGMT links predicted by SVDetect and NM links.** NM, INS_FRAGMT and INV_INS_FRAGMT links are colored in red, green and blue color gradients, respectively, with gradation according to the number of reads supporting the link. CNV is displayed in a heatmap style with color according to the CNV value computed by FREEC. Coverage is displayed in dark grey histogram style. Grey and dark green boxes correspond to the Watson and Crick gene annotations, respectively. ec and NM are the labels for the chromosome under study and the no-match pseudo-chromosome.

**Figure 4:**
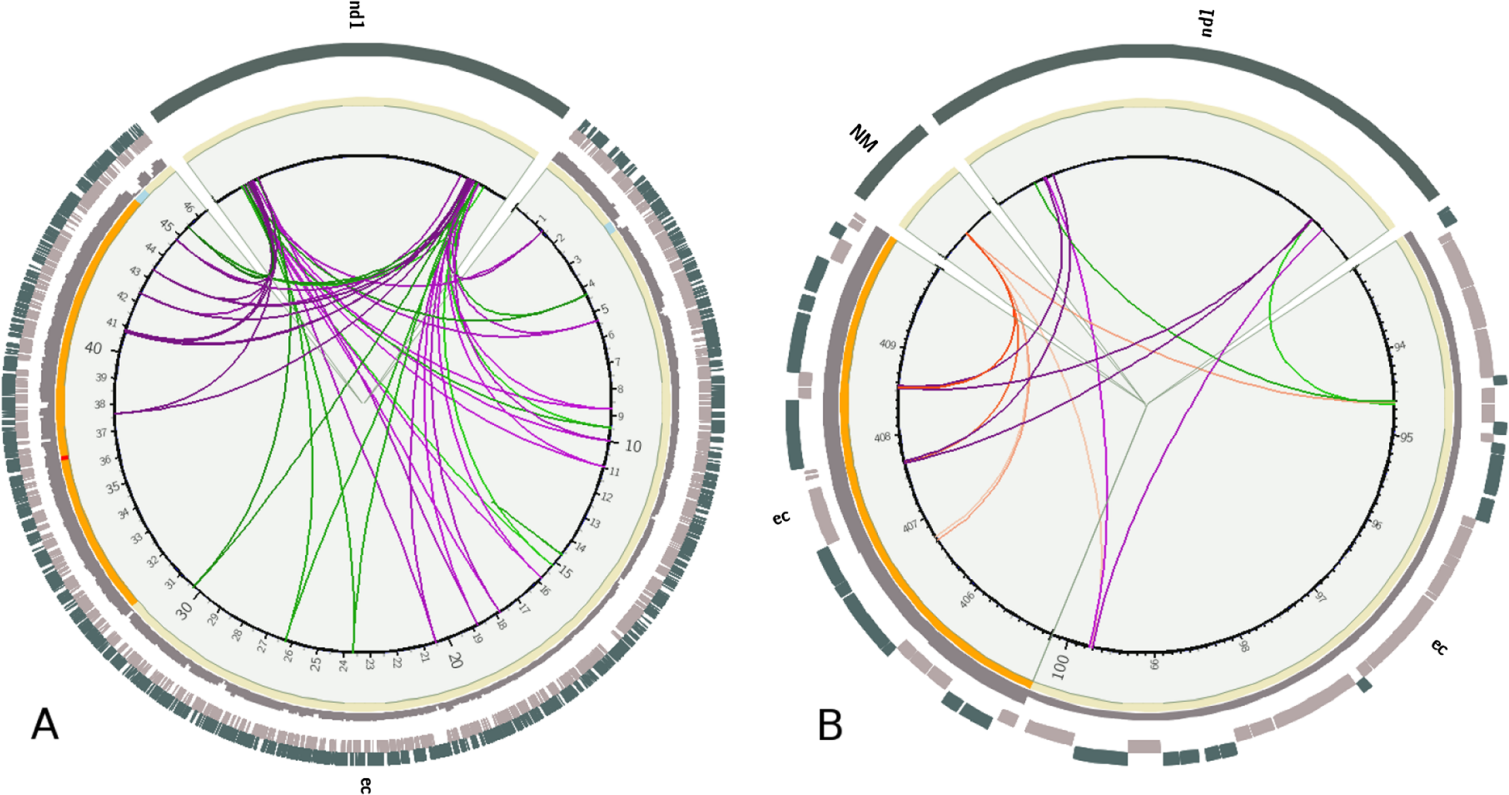
**CIRCUS images. (A)** Display of TRANSLOC and INV_TRANSLOC links between node1 and chromosome of interest and **(B)** zoom on two regions (between 930 kb and 1002 kb for one region and 4050 kb and 4100 kb for other region) for a more detailed analysis. NM, TRANSLOC and INV_ TRANSLOC links are colored red, green and pink gradients, respectively, with gradation according to the number of reads supporting the link. The contig is labeled nd1. Other labels are as in Figure 3.

To test CIRCUS, we have sequenced paired-ends from 2,958,998 genomic DNA fragments from an E. Coli strain on a GaIIx Illumina sequencer. A BAM file was created after mapping these reads with BWA on E. coli (K12_MG1655) as a reference genome. SVDetect was then used to predict variations between these two genomes. A quick display by CIRCUS of the INS_FRAGMT, INV_INS_FRAGMT and NM links (Figure 3) suggests multiple insertions of foreign sequences. Reads corresponding to fragments with no hit at both extremities were extracted from the BAM file and assembled by Velvet [8] (a sequence assembler for short reads). Overlapping reads were used to assemble synthetic continuous long reads (contigs). One of the resulting contigs corresponds to an enterobacteriophage transposase. After subsequent mapping of all the reads in this contig, and a prediction of genome variations by SVDetect, the display by CIRCUS of the TRANSLOC and INV_TRANSLOC links between the reference genome and the contig (referred to as nd1) shows a large number of transposase insertions (Figure 4A). A more precise localization in the gene landscape of some transposase insertion sites in two regions is presented to illustrate the ability of the package to analyze genomes at different scales (Figure 4B).

This study has been performed using the following functions, called mainly with default parameters.

Creating the karytotype file:

< create_karyotype (file = "K12_MG1655.fasta")

Computing reads coverage by bins of 10 kb:

< coverage_adapt (file = "CX1313.sam", win = 10000,

   chr_file = "karyotype.txt")

Adapting and coloring gene annotation:

< tab_annot_adapt (file = "K12_MG1655.gtf", coln = c(2,4,5,7,3))

< annot_paint (file = "K12_MG1655_annot.circus")

Processing and coloring NM links:

< NM_adapt (file = "CX1313.sam")

< NM_paint (file = "CX1313_NM.circus", fragSize = 330, threshold = 50)

Adapting CNV data from FREEC results:

< FREEC_CNV_adapt (file = "CX1313_CNVs")

< CNV_paint (file = "CX1313_CNVs_CNV.circus")

Adapting and coloring genome variations from SVDetect results:

< SVD_links_adapt (file = "CX1313.links.filtered")

< links_paint (file = "CX1313.links.filtered_links.circus",

   conv = data.frame(c("INS_FRAGMT", "INV_INS_FRAGMT"), c(2,3)))Producing the image of Figure 3:

< chromosome_image(chr = "K12_MG1655.fa", view1 = c(1,NA),

   chr_file = "karyotype.txt",

   feat_file = "K12_MG1655_annot_painted.circus", coverage = 1,

   NM_file = "CX1313_NM_painted.circus",

   links_file = "CX1313.links.filtered_links_painted.circus",

   CNV_file = "CX1313_CNV_painted.circus"   image_file = "Figure 3", ext_fraction = 5)Producing the image of Figure 4A:

< links_paint (file = "CX1313contig.links.filtered_links.circus",

   conv = data.frame(c("TRANSLOC", "INV_TRANSLOC"), c(2,4)))

< chromosome_image (chr = "K12_MG1655.fa", view1 = c(1, NA),

   chr_file = "karyotype.txt",

   feat_file = "K12_MG1655_annot_painted.circus", coverage = 1,   image_file = "Figure 4A",

   links_file = "CX1313contig.links.filtered_links_painted.circus",

   CNV_file = "CX1313_CNV_painted.circus")Producing the image Figure 4B:

< chromosome_image(chr = "K12_MG1655.fa", view1 = c(930000, 1002000),

   view2 = c(4050000, 4100000), chr_file = "karyotype.txt",   feat_file = "K12_MG1655_annot_painted.circus", coverage = 1, image_file = "Figure 4B",

   links_file = "CX1313contig.links.filtered_links_painted.circus",

   NM_file = "CX1313_NM_painted.circus", flag_view_outer = FALSE,

   CNV_file = "CX1313_CNV_painted.circus")

## Conclusions

The CIRCUS package is a simple solution for both biologists and bio-informaticians that want to display structural variants of genomes. As CIRCUS allows a programmer to easily add adaptors, its canvas may also be suitable for other applications, such as Hi-C, as long as events can be represented by links.

## Availability and requirements

CIRCUS is available at https://www.imagif.cnrs.fr/plateforme-36-Plateforme_de_Sequencage_a_Haut_Debit.html.

CIRCUS is an R package and requires the installation of the Circos software. It may also require the SAMtools and BEDtools packages as well as Python to allow reads coverage and NM links displays.

## Competing interests

The authors declare that they have no competing interests.

## Authors’ contributions

DN designed and implemented the software package, and wrote the manuscript. YdAC designed the package and drafted the manuscript. MS contributed to the improvement of the package and wrote the package tutorial. CT revised the manuscript. All authors read and approved the final manuscript.
